# Application of Plasma-Printed Paper-Based SERS Substrate for Cocaine Detection

**DOI:** 10.3390/s21030810

**Published:** 2021-01-26

**Authors:** Rhiannon Alder, Jungmi Hong, Edith Chow, Jinghua Fang, Fabio Isa, Bryony Ashford, Christophe Comte, Avi Bendavid, Linda Xiao, Kostya (Ken) Ostrikov, Shanlin Fu, Anthony B. Murphy

**Affiliations:** 1 Centre for Forensic Science, University of Technology Sydney, Sydney, NSW 2007, Australia; Rhiannon.L.Alder@student.uts.edu.au (R.A.); Linda.Xiao@uts.edu.au (L.X.); Shanlin.Fu@uts.edu.au (S.F.); 2 IDEAL ARC Research Hub, University of Technology Sydney, Sydney, NSW 2007, Australia; 3 CSIRO Manufacturing, Lindfield, NSW 2070, Australia; edith.chow@csiro.au (E.C.); fabio.isa@csiro.au (F.I.); bryony.ashford@csiro.au (B.A.); christophe.comte@csiro.au (C.C.); avi.bendavid@csiro.au (A.B.); tony.murphy@csiro.au (A.B.M.); 4 Aloxitec Pty Ltd., Lindfield, NSW 2070, Australia; aloxitec@gmail.com; 5 School of Chemistry, Physics and Mechanical Engineering, Queensland University of Technology, Brisbane, QLD 4001, Australia; kostya.ostrikov@qut.edu.au

**Keywords:** cocaine detection, plasma printing, SERS, gold nanoparticles, forensics, illicit drugs, on-site testing, paper substrate

## Abstract

Surface-enhanced Raman spectroscopy (SERS) technology is an attractive method for the prompt and accurate on-site screening of illicit drugs. As portable Raman systems are available for on-site screening, the readiness of SERS technology for sensing applications is predominantly dependent on the accuracy, stability and cost-effectiveness of the SERS strip. An atmospheric-pressure plasma-assisted chemical deposition process that can deposit an even distribution of nanogold particles in a one-step process has been developed. The process was used to print a nanogold film on a paper-based substrate using a HAuCl_4_ solution precursor. X-ray photoelectron spectroscopy (XPS) analysis demonstrates that the gold has been fully reduced and that subsequent plasma post-treatment decreases the carbon content of the film. Results for cocaine detection using this substrate were compared with two commercial SERS substrates, one based on nanogold on paper and the currently available best commercial SERS substrate based on an Ag pillar structure. A larger number of bands associated with cocaine was detected using the plasma-printed substrate than the commercial substrates across a range of cocaine concentrations from 1 to 5000 ng/mL. A detection limit as low as 1 ng/mL cocaine with high spatial uniformity was demonstrated with the plasma-printed substrate. It is shown that the plasma-printed substrate can be produced at a much lower cost than the price of the commercial substrate.

## 1. Introduction

With the availability of portable Raman systems, there is an enormous opportunity to create low-cost, highly sensitive and reliable surface-enhanced Raman spectroscopy (SERS) strips for on-site testing of trace illicit drugs [1,2,3,4,5] and explosives [6,7]. The inelastic scattering of photons by incident light can be used to determine the vibrational modes of molecules and thus provide a structural fingerprint of the molecule. Compared to conventional Raman techniques [8], the use of a roughened metal or metal nanoparticle surface in SERS enhances the Raman effect by typically 6–8 orders of magnitude [5] owing to localised surface plasmonic resonances around the surface protrusions or particles [9]. Fedick et al. developed an undergraduate experiment that incorporated measurements using a commercial silver-on-paper SERS substrate for the detection of heroin, fentanyl and 3,4-methylenedioxymethamphetamine [10]. Inkjet printing methods have allowed the construction of SERS substrates that can be used for two-dimensional chromatographic separation for complex matrix analysis [11]. These inkjet-printed substrates allowed quantification of 25 ng of heroin mixed with highly fluorescent materials [11]. Silver nanoparticle-soaked filter paper discs were used by Haddad et al. for the analysis of fentanyl-spiked heroin [1]. The limit of detection for fentanyl was 100 ng/L when 10 µL of analyte solution was deposited. Swabbing with these substrates allowed the recovery of fentanyl from surfaces.

Despite the advances in SERS testing, there remains an opportunity to improve the analyte sensitivity and reduce the complexity in the fabrication of SERS-active substrates. Optimisation of the shape and size distribution of the nanometals allows enhancement of the Raman signal from analyte molecules. Sophisticated shapes and combinations of nanometal particles have been intensively studied, such as nanoflowers [12], nanostars [13], sea-urchin-shaped nanoparticles [14], as well as different types of core-shell structure [15]. Various fabrication techniques have also been applied, including electron beam lithography [16], nanosphere lithography [17] and focused ion beam patterning [18], to achieve high sensitivity and reproducibility. However, most are elaborate multi-step processes that are costly and are not amenable for large-scale production and on-site or point-of-care testing.

The choice of substrate and materials deposition technique are two other important considerations for SERS-based applications. Paper-based substrates are highly attractive as they are low-cost, disposable and can be readily modified by inkjet printing, drop-casting, direct writing and soaking with different nanomaterials [19,20,21,22,23]. Moreover, paper-based substrates are flexible, which could allow for swabbing applications [24,25] within the forensics field for detection of illicit drugs and explosives.

Inkjet printing [26,27,28] provides the best control over the uniformity of gold nanoparticle films with microscale precision and is amenable to high-throughput, rapid-prototyping of SERS substrates. However, the preparation of the ink requires several steps, including metal nanoparticle synthesis and filtering [29]; alternatively, commercial nanogold or nanosilver inks are available but costly. There is also the possibility of interference from residual chemicals such as reducing or stabilising agents in SERS measurements of low concentrations of analyte molecules.

Alternative methods that can reduce the fabrication complexity of nanogold substrates are highly desirable. Plasma deposition, especially atmospheric-pressure plasma-assisted chemical vapour deposition (CVD), has recently been demonstrated as a facile and cost-effective processing technique for nanogold deposition [30]. The advantage of this technique is that it is possible to produce and deposit nanogold films directly on the substrate from a single HAuCl_4_ solution precursor without additional reducing or stabilising agents. Furthermore, there is no need for multi-step filtration, centrifugation or purification steps that are commonly employed in nanoparticle synthesis. A plasma jet of high-density electrons reduces the solution to metallic gold, which is then deposited on the substrate almost instantaneously. Since plasma printing does not require complex and multiple chemical steps, nor high-end electron or ion beam processing under vacuum, it is a promising alternative technique to fabricate highly sensitive, low-cost paper-based SERS substrates.

In this study, a paper-based SERS substrate is produced by depositing nano gold on paper using the plasma-assisted CVD technique. The substrate is applied to the detection of cocaine. Cocaine, an alkaloid compound, is the second most-consumed stimulant in Australia [31] with approximately 4.1 tonnes consumed each year [32,33].

The main routes of administration include insufflation, smoking and injection with the administration route depending on the drug form. In Australia, cocaine hydrochloride is the most common form [32] and is administered by insufflation or rubbing on the gums [34,35]. Consumers under the influence of cocaine can exhibit behaviour that is unpredictable, violent or aggressive, which can be dangerous to both themselves and others [34,36]. Current on-site testing for cocaine involves colour testing or immunoassay strips, which then require confirmatory testing [37]. Ideally, a method that can provide rapid on-site testing is highly desirable.

The plasma-printed SERS substrates are compared to two commercially available substrates, a paper-based gold SERS substrate and a silver pillar structure on Si that presents the state-of-the-art for cocaine detection. The plasma-printed SERS substrate shows promise as a simple and scalable fabrication technique for the highly sensitive detection of cocaine.

## 2. Materials and Methods

### 2.1. Chemicals

Whatman No. 1 filter paper and gold(III) chloride trihydrate (HAuCl_4_·3H_2_O) were purchased from Sigma-Aldrich (Macquarie Park, Australia). Cocaine hydrochloride was purchased from the National Measurement Institute (West Lindfield, Australia). Ultra-pure Milli-Q water (>18.2 MΩ cm) and ethanol (Wilmar, Yarraville, Australia) were used for the preparation of solutions. Ar and He gases were purchased from BOC in 99.997% high purity and 99.999% ultra-high purity grades, respectively.

### 2.2. SERS Substrate Fabrication

**Figure 1:**
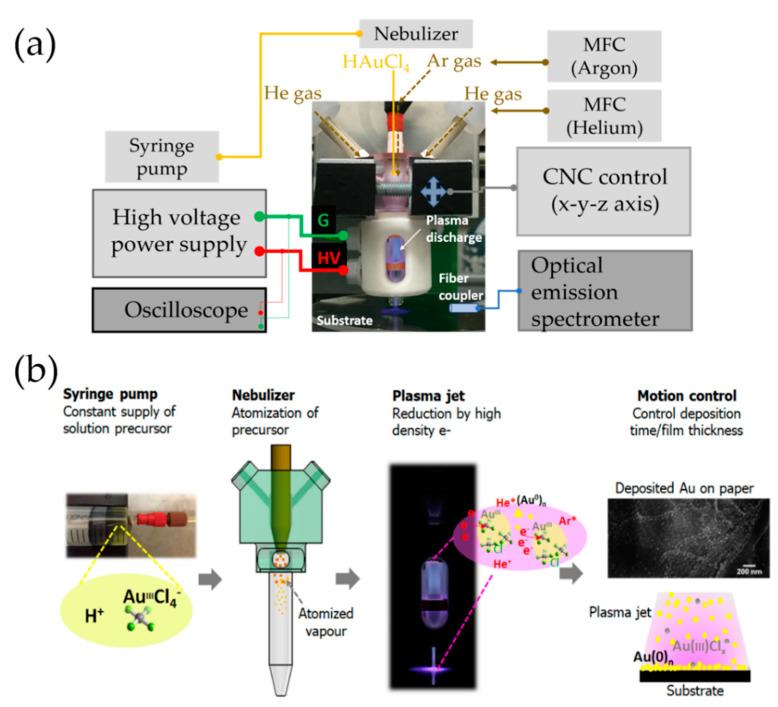
Experimental configuration (**a**) and illustration of the working principle and elements of the plasma jet printing process (**b**); adapted from Hong et al. [30] with permission from The Royal Society of Chemistry.

Nanogold was deposited on paper (Whatman filter paper No. 1) from an HAuCl_4_ precursor solution using the atmospheric-pressure plasma jet. 1% *w*/*v* HAuCl_4_ aqueous solution was prepared and mixed with ethanol in 1:1 volume ratio to provide improved atomisation. Using a syringe pump (Harvard PHD 2000), 20 µL/min of the liquid source was supplied to a parallel-path pneumatic nebuliser (Burgener Research Inc., Ontario, Canada), which atomised the droplet into a fine vapour through interaction with a fast Ar gas stream as shown in Figure 1a,b. The plasma jet consisted of a custom-blown glass tube (Pyrex glass, inner diameter 6 mm, thickness 1.5 mm) and two parallel ring shape electrodes. It was powered by a high-voltage AC power supply (PVM500) operated typically at 23 kHz with a peak voltage 7.0 ± 0.5 kV. Using a mass flow controller (SevenStar D08), 0.5 LPM of Ar and 4 LPM of He were supplied to the active plasma discharge region. The plasma jet module is situated on a table-top CNC (Computer Numerical Control) machine (High Z—cncstep.de) in order to deposit and print a specified pattern. The separation between the glass tube aperture and the substrate was 2 mm.

The scanning motion of the plasma jet was tested with different scanning times, with parameters chosen to optimise the film properties for highly sensitive SERS measurement. In this work, all samples were deposited with six passes, each of 3 mm width at 1 mm/s. The influence of the number of passes is shown in Figure S1 in the SI.

Unless otherwise noted, the deposited films were plasma post-treated. This was done by scanning the films twice with the same plasma jet with He gas only at the same input power conditions (23 kHz, peak voltage 7.0 ± 0.5 kV) and the scanning speed of 1 mm/s.

### 2.3. Plasma Characterisation

The optical emission spectra were measured using an optical emission spectrometer (Acton SP2500/Princeton Instrument). The slit width was 10 μm, and the exposure time was 3 s. A fibre input coupler placed at a radial distance of 20 mm from the plasma jet was used. Measurements were performed for both the active plasma discharge region at the same height as the midpoint between the two electrodes, and at a position 2 mm above the substrate.

### 2.4. Surface Characterisation

XPS measurements were performed using a Specs150 SAGE instrument with an Mg Kα X-ray source with energy 1253.6 eV. The resolution for the energy scale is 0.1 eV and 15 scans are accumulated for the elemental analysis.

The surface morphology of the printed nanogold film were characterized using field-emission scanning electron microscope (FE-SEM.; Zeiss) operated at electron beam energies of 5 keV with an InLens secondary electron detector.

### 2.5. Standard Dilutions

A stock solution of cocaine was prepared by dissolving 1 mg of solid cocaine hydrochloride powder in 1 mL of MilliQ water. Serial dilutions were performed to produce standards with concentrations of 5000, 1000, 500, 100, 10 and 1 ng/mL. A 5 µL aliquot was deposited onto the SERS substrate [38].

### 2.6. Oral Fluid Extractions

Oral fluid was collected under human ethics approval No: UTS ETH18–2521. Oral fluid was spiked at concentrations of 10 and 100 ng/mL. Spiked and blank oral fluid samples (100 µL) were pH adjusted with 100 µL 0.1 M pH 9.2 carbonate buffer and extracted with 100 µL 9:1 dichloromethane (DCM): isopropyl alcohol (IPA) [38]. The extraction method was adapted from Clauwaert et al. [39]. A 5 µL aliquot of the organic phase was deposited onto the plasma-printed substrate.

### 2.7. Raman Analysis

Raman analysis was conducted using a Renishaw inVia Raman microscope with 785 nm laser and 1200 line/mm grating. The analysis was conducted with a laser power of 20 mW, over the range of 550–2000 cm^−1^ with 10 s exposure, single accumulation and pinhole in. The microscope objective was set to 20× magnification. For the detection of different concentrations of cocaine, 20 spectra were collected across different points on the substrate surface [38].

Raman mapping involved constructing a montage of the microscope images across the entire surface and taking consecutive measurements within a grid. The distribution of compounds on a surface was determined by picking characteristic bands and having the image displayed as an intensity heat map. Mapping was set up over the still image montage of the entire surface of the substrate. The montage was constructed using eight images in the x-direction and 13 images in the y-direction. Mapping steps were 175 µm × 175 µm in a grid for a total of 399 spectra collected across the surface. The mapping review was conducted using the intensity at a point across four common cocaine bands, 1003 cm^−1^, 1032 cm^−1^, 1450 cm^−1^ and 1600 cm^−1^.

### 2.8. Comparison with Commercial SERS Substrates

Commercial gold paper-based SERS substrates (P-SERS) were purchased from Metrohm Australia Pty Ltd. (Sydney, Australia) and silver-coated silicon pillar substrates were purchased from JASMAT Optics Corp (Taiwan). Cocaine standard solutions ranging in concentration from 1–5000 ng/mL were used to compare the SERS spectra of the plasma-printed and commercial substrates. The visible cocaine vibrational bands were annotated on the spectra and tabulated.

## 3. Results

### 3.1. Plasma Characterisation

The estimated average power density in the plasma was 4.0 ± 0.3 W/cm^3^, calculated using an estimated discharge volume of 1.6 cm^3^. The average electron number density was calculated to be (1.4 ± 0.2) × 10^10^ cm^−3^, as has been previously reported [30]. The optical emission spectrum was measured to understand the plasma reduction process in the active discharge zone between the electrodes and near the substrate. The emission spectra from various excited states of molecules, radicals, ions and atoms were observed, indicating a highly reactive environment of the plasma discharge with a low gas temperature of 360 ± 30 K. The detailed optical emission measurement results are provided in the SI. Unlike the high-temperature N_2_ plasma with a large amount of chloroauric acid on the surface presented by Wu et al. [40], no excited AuCl molecules were observed. Maguire et al. [41] suggested the high density of electrons in the plasma discharge may provide a rapid reduction of HAuCl_4_. Therefore, AuCl emission may not be observable because the lifetime of AuCl will be very short in a high-density plasma discharge with finely atomised vapour.

### 3.2. Surface Characterisation

**Figure 2:**
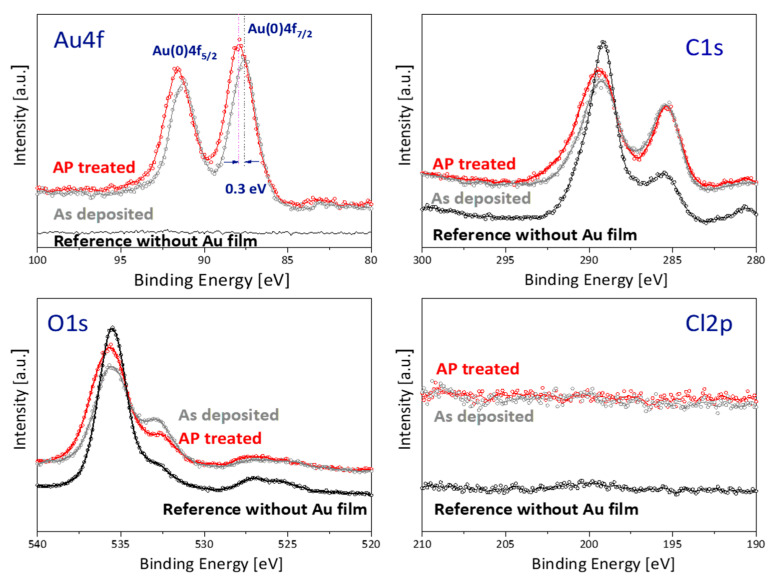
Elemental XPS spectra of Au4 f, C1 s, O1 s and Cl2 p for plasma-printed nanogold film before (‘as deposited’) and after (‘AP treated’) plasma post-treatment and the reference case of the paper base without gold film.

SEM images, shown in Figure S2 in the SI, revealed uniform deposition of nanogold particles along the intrinsic matrix of the paper substrate. However, due to the charging problem, it was not possible to investigate the detailed structure and shape at high magnification. The performance of the deposited nanogold film as a SERS substrate was greatly improved by plasma post-treatment. Only plasma post-treated substrates were able to detect cocaine. They also showed significantly improved sensitivity in detecting low concentrations of Rhodamine B, as shown in Figures S3 and S4 in the SI. The improvement is attributed to the reduction of amorphous carbon content in the nanogold films. The carbon introduced by ethanol dissolved in the precursor solution. As we described in Section 2.2, the plasma post-treatment was done using a He plasma jet at atmospheric pressure. Because it is operated under ambient conditions, it can interact with the molecules in the surrounding air and generate reactive radicals. It is expected to introduce new functional groups and modify the surface properties, as is commonly reported for many plasma processes at atmospheric pressure. A decreased C-C bond and newly introduced oxygen functional groups are commonly observed when vacuum plasmas containing oxygen or ambient-air-exposed atmospheric-pressure plasmas are used to treat carbon-based organic materials such as fibres or polymeric substances [42,43]. Figure 2 shows the XPS spectra of Au4 f, C1s, O1s and Cl2p for the nanogold film before and after plasma post-treatment. The post-treatment causes a 0.4 eV shift in the Au4f peak and a clear increase in the O1 s peak intensity. The atomic composition of the nanogold film, obtained using SpecsLab analysis software, is given in Table 1, where the instrumental error, including variation of X-ray intensity, analyser pass energy, aperture settings, etc., is known to be at most. 1% for C or O, and is significantly lower for elements such as Au and Cl. The content of Cl was below the detection limit before and after post-treatment, indicating a high level of reduction of the ionic gold in the precursor. As shown in Figure 2, the oxygen content was increased, and the carbon content decreased, by the plasma post-treatment.

Table 2 shows a comparison of the components of the C1 s peak for as-deposited and post-treated nanogold films with the components of the peak for the paper-based substrate without deposited gold. Casa XPS software was used to deconvolute the peaks with max. 0.5% error. The results indicate that plasma post-treatment has removed the amorphous carbon layer and increased mainly the amount of O-C=O bonds.

### 3.3. Cocaine Analysis

**Figure 3:**
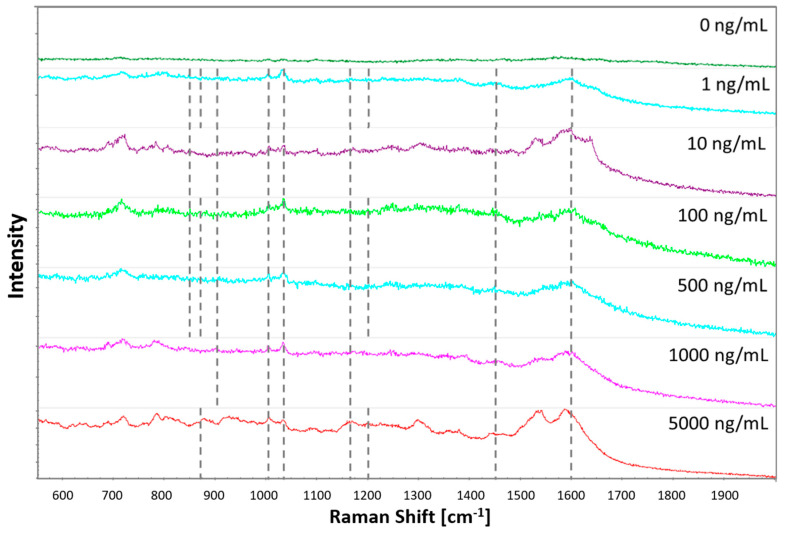
SERS spectra of cocaine standards of increasing concentration deposited onto plasma‒printed substrates.

Figure 3 shows the visible cocaine vibrational bands when tested on the plasma-deposited nanogold substrate after post-treatment, with increasing cocaine standard concentrations. It shows that the plasma-printed SERS substrate allows the detection of six to nine characteristic cocaine vibration bands. The bands and the corresponding vibration modes are listed in Table 3. Five bands, at 1003 cm^−1^, 1032 cm^−1^, 1164 cm^−1^, 1450 cm^−1^ and 1600 cm^−1^, were consistently enhanced across the concentrations tested. These bands correspond to the symmetric and asymmetric ring breathing, C-N stretching, asymmetric -CH_3_ deformation and C=C aromatic ring stretching, respectively. Furthermore, at least one of the three C-C tropane ring stretching bands between 848–900 cm^−1^ was observed for each concentration. The band at 1200 cm^−1^, corresponding to the other C-N stretching band, was observed at concentrations of 1, 100, 500 and 5000 ng/mL.

### 3.4. Spatial Distribution

**Figure 4:**
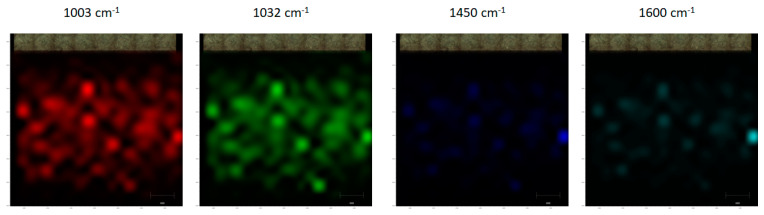
Raman heat maps showing the spatial distribution of cocaine using the four characteristic bands. The mapping steps were 175 µm × 175 µm in a grid for a total of 399 spectra collected across the surface area 3 mm × 3 mm.

The consistency of the enhancement across the plasma-printed substrate was determined using Raman mapping with four common cocaine band intensities. The intensity heat maps shown in Figure 4 have a good correlation of the intensities across the four bands. The distribution across the entire surface of the substrate was found to be consistent except along the edges where the deposited surface was no longer visible. The intensity across these four bands allows one to conclude that the hotspots were distributed across the surface resulting in surface-wide detection.

### 3.5. Comparison with Commercial SERS Platform

The commercial substrates compared to the developed plasma printed substrate were a paper-based SERS (P-SERS) substrate with the gold SERS active metal deposited through inkjet printing and a silicon pillar-based substrate coated in silver (JASMAT Ag). These substrates were chosen for comparison as the P-SERS had a similar composition to the plasma-printed substrate and the JASMAT Ag had previously been shown to be effective for cocaine analysis [38].

**Figure 5:**
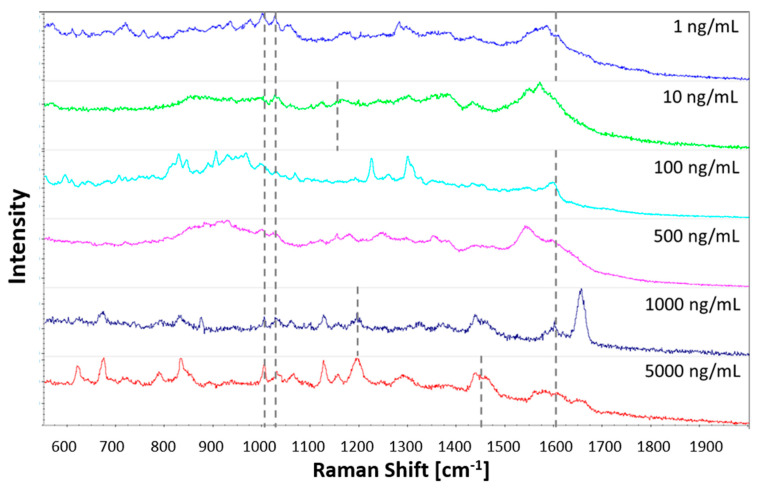
SERS spectra collected from cocaine deposited on commercial P‒SERS substrate.

Figure 5 shows Raman spectra measured on commercial P-SERS substrate with increasing cocaine standard concentrations. Only two Raman bands were consistently enhanced on the commercial substrate at ~1000 cm^−1^ and ~1027 cm^−1^ in the presence of cocaine. These correspond to the symmetric and asymmetric stretching of the aromatic ring. The band at ~1600 cm^−1^ from C=C aromatic stretching was observed for all the concentrations except the 10 ng/mL standard. The tropane and carbonyl bands were not observed in any of the samples. The C-N stretching band at ~1162 cm^−1^ was only observed once at 10 ng/mL concentration, while the second C-N stretching band at ~1198 cm^−1^ was only observed at a concentration of 1000 ng/mL and the asymmetric -CH_3_ deformation band at ~1446 cm^−1^ was observed at 5000 ng/mL. When compared to the developed plasma deposited substrate results, this commercial substrate enhanced fewer cocaine vibrational bands at each concentration. Furthermore, only two bands were enhanced across the tested concentrations compared to five consistent bands on the developed substrate. For the detailed information on each different vibrational band detected on P-SERS substrate, see Table S1.

The commercial P-SERS substrate did have a more intense peak at both of the consistently enhanced bands. However, these bands are common among the drugs tested as they correspond to aromatic ring breathing bands. Therefore, vibrational bands need to be consistently enhanced to produce a characteristic fingerprint of the analyte. The analyte can only be confirmed if enough of the characteristic bands are visible.

**Figure 6:**
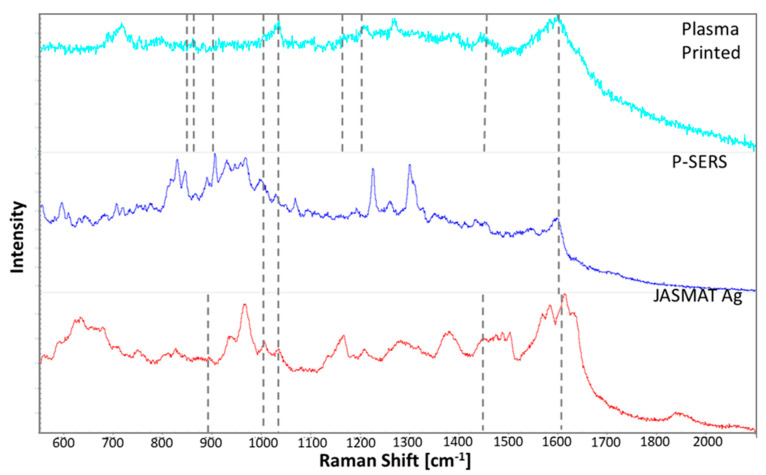
Comparison of cocaine at 100 ng/mL concentration deposited onto the plasma printed substrate and two commercial SERS substrates, P‒SERS and JASMAT Ag.

The three substrates were compared using the number of bands enhanced at a concentration of 100 ng/mL as shown in Figure 6, and the number of bands consistently enhanced across the six concentrations, presented in the SI. The plasma-printed substrate enhanced between six and nine bands for each concentration. Five of these bands were consistently enhanced across all of the concentrations. The commercial P-SERS only enhanced three or four bands, with only two consistently enhanced. The commercial JASMAT Ag substrate enhanced between five and seven bands for cocaine, with four being consistently enhanced as shown in Figure S8 and Table S2 of SI. At the concentration of 100 ng/mL, shown in Figure 6, the plasma-printed substrate enhanced nine cocaine bands. The commercial P-SERS and JASMAT Ag substrates enhanced three and five bands, respectively. The cocaine bands tend to be more intense for the commercial substrates. The plasma-printed substrate outperformed the two commercial substrates for the analysis of cocaine based on both the number of enhanced bands and number of consistently enhanced bands.

### 3.6. Application to Oral Fluid

**Figure 7:**
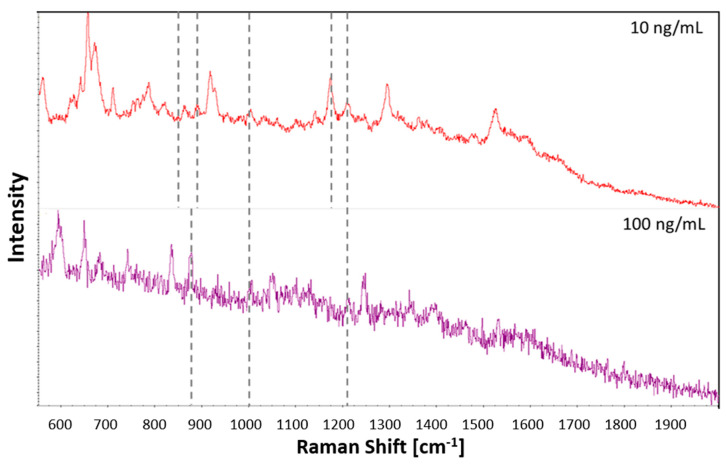
Comparison of SERS spectra acquired from extracted cocaine spiked oral fluid at 10 ng/mL and 100 ng/mL deposited onto the plasma printed substrate.

The plasma-printed substrate was tested with cocaine extracted from oral fluid spiked at cocaine concentrations of 10 ng/mL and 100 ng/mL. The results are presented in Figure 7. At 100 ng/mL, there were three visible cocaine bands. These correspond to (C-C) stretching of the tropane ring, symmetric aromatic ring breathing and C-N stretching. The lower concentration of 10 ng/mL revealed five cocaine bands. The enhanced bands corresponded to the (C-C) stretching of the tropane ring at 850 cm^−1^ and 897 cm^−1^, symmetric aromatic ring breathing at 1003 cm^−1^, and C-N stretching at 1164 cm^−1^ and 1205 cm^−1^. Due to the possible interference from many other compounds and proteins in oral fluid, a lower number of enhanced bands was visible than in the standard of the same concentration. However, it is an important result to demonstrate the possible application of the plasma printed SERS strip in real application of on-site illicit drug testing.

### 3.7. Cost Comparison

To demonstrate the cost-effectiveness of the plasma process, the production cost of the plasma-printed nanogold SERS substrate is estimated and compared to the price of the two commercial strips in Table S3 in the SI. The costs of electricity, gas, liquid precursor, filter paper and depreciation of the equipment such as power supply, liquid pump, mass flow meters and nebuliser are included based on the assumption of a five-year lifetime. The labour cost or other possible indirect expenses are not included. The total estimated cost to produce a single SERS substrate using the current plasma system is 0.107 AUD. This is doubled to take into account possible errors or interruptions in processing. The details of the cost estimation are given in Table S4 of the SI. For the cost calculation, the active area is presumed to be the same as that of the commercial JASMAT Ag substrate. However, the plasma-printing process enables continuous processing, unlike batch processes such as e-beam evaporation or sputtering, which also require an expensive high vacuum system. For large-scale processing, the current single jet system could be redesigned into an array or slit jet to cover a large area at the same time. With that modification, a further decrease in cost can be expected because liquid pumps and gas flow meters can be shared.

## 4. Discussion

Plasma-printed nanogold on a paper based was demonstrated to be a highly sensitive and cost-effective SERS substrate for cocaine detection. The plasma-printed substrate was able to detect between six and nine characteristic Raman peaks of cocaine at concentrations from 1 to 5000 ng/mL, whereas a commercial SERS gold on a paper-based substrate enhanced only three to four bands. In addition, the plasma-printed SERS substrate provided better consistency than the commercial SERS substrate with Ag pillar structure, which is currently favoured due to its high sensitivity. It is likely that the direct plasma deposition of nanogold from a solution precursor provides desirable surface conditions without interference from residual chemicals, such as reducing or stabilising agents. The additional plasma post-treatment step removed the organic carbon layer, which may have formed as a result of the ethyl alcohol used as a diluting solvent to improve the atomisation of the precursor. The paper-based plasma-printed SERS substrate has the potential to be a practical, economical solution for on-site screening or point-of-care applications, such as illicit drug detection, when used in combination with a portable Raman system.

## Figures and Tables

**Table 1:** Atomic composition of nanogold film deposited on paper, measured by XPS.

	C [at %]	O [at %]	Au [at %]	Cl [at %]
As deposited	63.9	34.1	2.0	0.0
Post-treated	59.6	38.3	2.1	0.0

**Table 2:** Influence of plasma post-treatment on deposited nanogold XPS signals: comparison of components of C1s peak.

C1s.	Binding Energy [eV]/Composition [%]			
	C-C	C-O	C=O	O-C=O
Paper base	285.0 eV (21.1%)	286.7 eV (4.0%)	288.5 eV (65.5%)	290.2 eV (9.4%)
As deposited	284.8 eV (35.2%)	286.5 eV (5.7%)	288.6 eV (49.1%)	290.1 eV (10.1%)
Post-treated	284.8 eV (32.0%)	286.7 eV (2.8%)	288.7 eV (49.1%)	290.3 eV (16.1%)

**Table 3:** Cocaine bands detected in cocaine standards on plasma-printed substrate.

Bands Listed in Ref. [3]		Plasma-Printed Substrate						
Vibration Mode	Cocaine HCl Salt	Cocaine HCl Salt	5000 ng/mL	1000 ng/mL	500 ng/mL	100 ng/mL	10 ng/mL	1 ng/mL
(C-C) stretching (tropane ring)	848 874 898	853 870 897	874	898	850 874 901	850 873 899	852 898	855 872 900
Symmetric stretching-aromatic ring breathing	1004	1001	1004	1005	1003	1004	1005	1004
Asymmetric stretching-aromatic ring breathing	1026	1027	1028	1027	1029	1026	1028	1027
C-N stretching	1165	1164	1168	1168	1164	1168	1170	1169
C-N stretching	1207	1205	1201	-	1208	1203	-	1202
Asymmetric CH_3_ deformation	1462	1459	1455	1458	1454	1460	1455	1458
C=C stretching-aromatic ring	1596, 1601	1599	1595	1600	1601	1602	1599	1600
C=O symmetric stretching-carbonyl	1716	1717	-	-	-	-	-	-
C=O asymmetric stretching-carbonyl	1735	-	-	-	-	-	-	-

## Data Availability

Data sharing not applicable.

## Supplementary Materials

The following are available online at https://www.mdpi.com/1424-8220/21/3/810/s1, Figure S1: Influence of the number of nanogold deposition passes on SERS performance, Figure S2: SEM images of plasma printed nanogold film on paper substrate for SERS measurement, Figure S3: Comparison of SERS spectra of nanogold substrates with and without post-treatment, Figure S4: Comparison of SERS spectra of nanogold substrates with and without post-treatment using 10^−6^ M of Rhodamine B aqueous solution, Figure S5: Optical emission spectra in the range of 200–800 nm, Figure S6: Optical emission from different species in the active discharge region, Figure S7: Optical emission from different species near the substrate and Figure S8: Comparison of SERS spectra acquired from standard cocaine solutions with decreasing concentrations deposited onto commercial JASMAT Ag; Table S1: Cocaine bands detected in cocaine standards on the commercial P-SERS substrate, Table S2: Cocaine bands detected in cocaine standards on the JASMAT Ag substrate, Table S3: Comparison of SERS substrates showing nanoparticle type, backing substrate, deposition technique, particle size, size of active area and cost per substrate and Table S4: Estimated cost of SERS strip printing using plasma jet based on current lab-scale system.
