# Supplementary material for: Application of Plasma-Printed Paper-Based SERS Substrate for Cocaine Detection
Source: Sensors (Basel). 2021 Jan 26;21(3):810. doi: 10.3390/s21030810 (PMC7866229; doi:10.3390/s21030810)
Supplement: Supplementary file 1 [file sensors-21-00810-s001.pdf]

Application of Plasma-Printed Paper-Based SERS Substrate for Cocaine Detection

Rhiannon Alder [1,2,†], Jungmi Hong [3,*,†], Edith Chow [3], Jinghua Fang [4], Fabio Isa [3], Bryony Ashford [3], Christophe Comte [3], Avi Bendavid [3], Linda Xiao [1], Kostya (Ken) Ostrikov [5], Shanlin Fu [1,2] and Anthony B. Murphy [3]

[1] Centre for Forensic Science, University of Technology Sydney, Sydney NSW 2007, Australia; Rhiannon.L.Alder@student.uts.edu.au (R.A.); Linda.Xiao@uts.edu.au (L.X.); Shanlin.Fu@uts.edu.au (S.F.)
[2] IDEAL ARC Research Hub, University of Technology Sydney, Sydney NSW 2007, Australia
[3] CSIRO Manufacturing, Lindfield NSW 2070, Australia; edith.chow@csiro.au (E.C.); fabio.isa@csiro.au. (F.I.); bryony.ashford@csiro.au (B.A.); christophe.comte@csiro.au (C.C.); avi.bendavid@csiro.au (A.B.); tony.murphy@csiro.au (A.B.M.)
[4] Aloxitec Pty Ltd., Lindfield NSW 2070, Australia; aloxitec@gmail.com
[5] School of Chemistry, Physics and Mechanical Engineering, Queensland University of Technology, Brisbane QLD 4001, Australia; kostya.ostrikov@qut.edu.au
* Correspondence: jungmi.hong@csiro.au
† These authors contributed equally.

Supplementary Information

1. Effect of nanogold thickness on SERS sensitivity

Four different nanogold substrates were prepared by scanning the plasma jet at a speed of 1 mm/s for 2, 4, 6 and 8 passes. Increasing the number of passes gives a denser and thicker gold film. Figure S1 shows the SERS spectra for each substrate after plasma post-treatment and the deposition of 5000 ng/mL cocaine. Only the substrates prepared using 6 and 8 passes allowed the detaction of cocaine. All other SERS measurements presented were performed using substrates prepared with 6 passes.

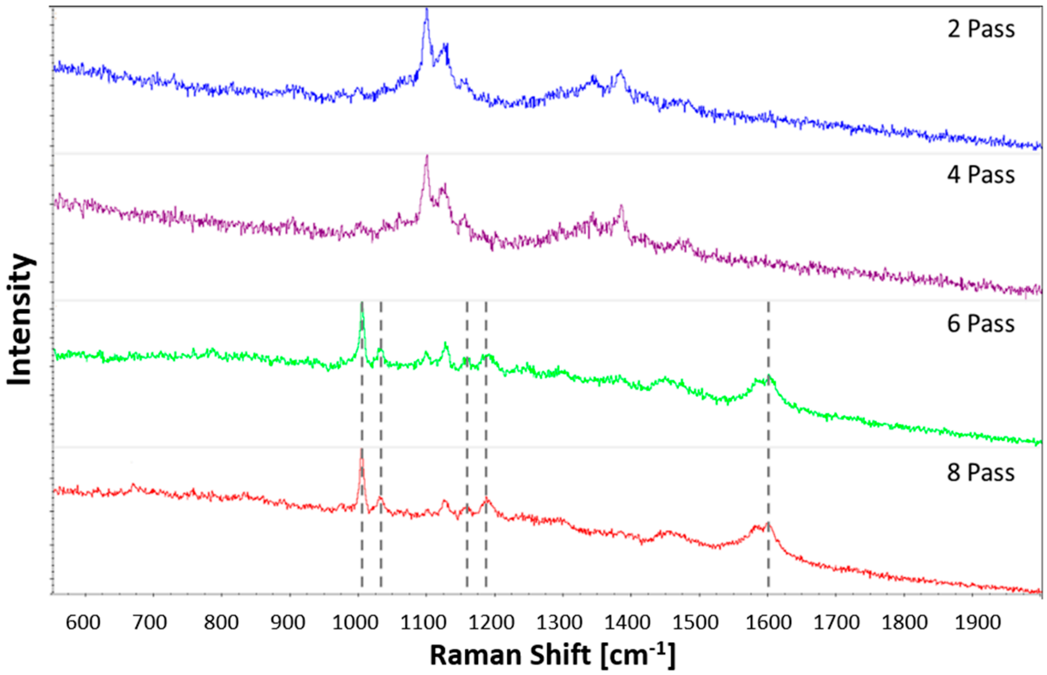

Figure S1. Influence of the number of nanogold deposition passes on SERS performance for the detection of 5000 ng/mL cocaine. Plasma post-treatment was applied to all samples.

2. SEM images of plasma printed nanogold on paper substrate

The surface morphology of the deposited nanogold film was characterized by SEM, as shown in **Error! Reference source not found.**At the macroscale, the deposited gold film shows uniform coverage of nanogold particles following the intrinsic 3D matrix of paper platform. The concentration of the solution of the HAuCl4 precursor, plasma power and gas flow rate can influence the physical and chemical properties of deposited gold film. The deposition time, which is altered by varying the number of scans of the plasma jet, was a crucial factor to optimize the size distribution and number density of the deposited nanogold in order to improve SERS sensitivity, as was shown with HIM (Helium Ion Microscopy) measurements in our previous work [1]. In this study, a larger exit plasma discharge tube (diameter 3 mm) has been used than previously (diameter 2 mm). The optimized SERS results were obtained at a higher number of passes, 6 or 8 passes as shown in Fig. S1, than the previous optimum of 4 passes, due to the decreased spatial density of the deposited nanoparticles.

In order to perform surface-oriented imaging and minimize the surface charging, the accelerating voltage was limited to 5 kV. However, severe charging persisted and prevented the acquisition of high quality images at high magnification. Therefore, it was difficult to distinguish and compare the surface morphology of printed gold before and after the plasma post-treatment.

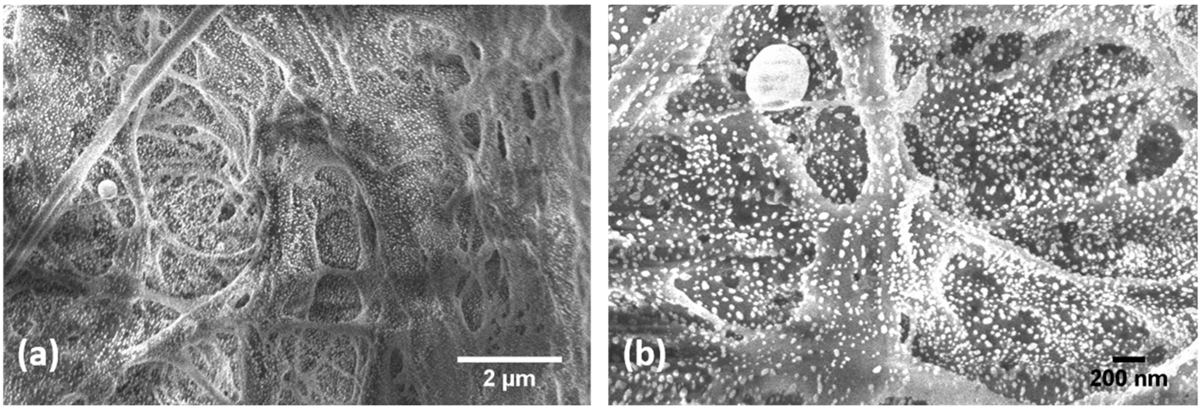

Figure S2. SEM images of plasma printed nanogold film on paper substrate for SERS measurement

3. Improved SERS sensitivity by plasma post-treatment

The substrates that were not post-treated were not able to detect cocaine, as shown in **Error! Reference source not found.**The addition of 5000 ng/mL cocaine on a nanogold substrate that was not post-treated did not make any discernible difference to the spectrum. An enhanced SERS signal from cocaine was only observed on the plasma post-treated substrate. It is also supported by significantly enhanced sensitivity in detection of Rhodamine B. As shown in Figure S4 two different plasma deposited samples were tested and showed significantly increased intensity of characteristic Raman peaks after plasma post-treatment.

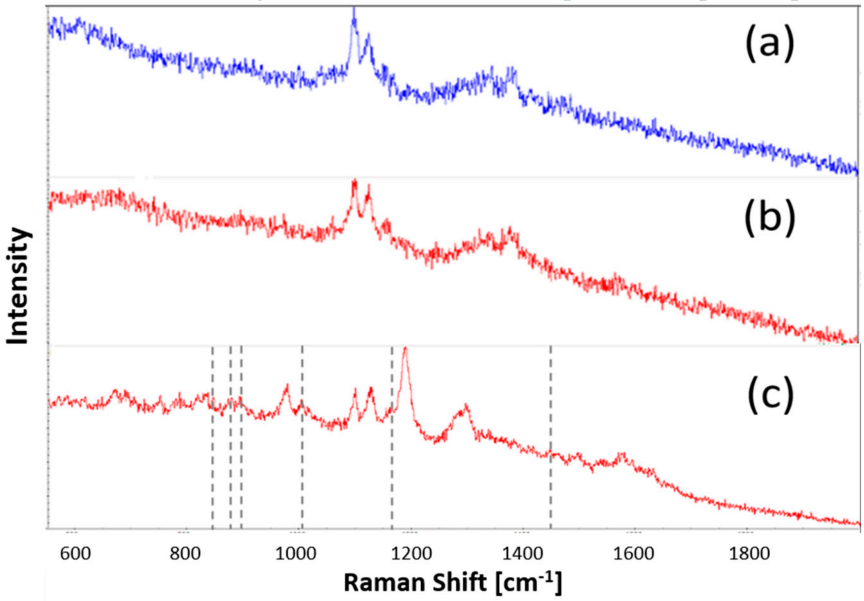

Figure S3. Comparison of SERS spectra of nanogold substrates with and without post-treatment; (a) non-post-treated substrate without cocaine, (b) non-post-treated substrate with 5000 ng/mL cocaine, (c) post-plasma treated substrate with 5000 ng/mL cocaine.

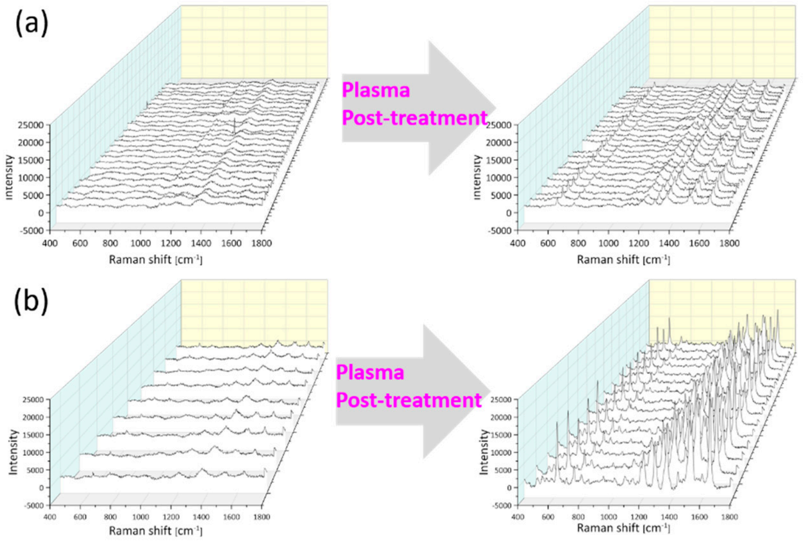

Figure S4. Comparison of SERS spectra of nanogold substrates with and without post-treatment using 10^{-6} M of Rhodamine B aqueous solution; deposited nanogold film on paper with one pass (a) and four passes (b) using exit diameter 1.5 mm tube, where Raman analysis was conducted using the Renishaw inVia Raman microscope with 633 nm laser source with 1200 l/mm grating.

4. Optical emission spectroscopy results

The measured emission spectra revealed the existence of various excited states of molecules, radicals, ions and atoms in the active plasma discharge region and also near the substrate, as shown in Figures S5 to S7. Figure S5(a) shows the dominant atomic Ar emission, strong emission from OH radicals and relatively weak emission from nitrogen molecules in the main discharge area. In contrast, near the substrate, stronger emission from nitrogen molecules can be observed. Because the plasma jet is operated under ambient conditions, it can interact with and excite the molecules in the surrounding air, as is commonly reported in many plasma processes at atmospheric pressure. A detailed mechanism of how those electronically excited nitrogen molecules can influence the reduction of $HAuCl_4$ solutions is not known. However, Wu et al. suggested rapid reduction of chloroauric acid on a surface is possible due to the high reactivity of atmospheric-pressure N_2 plasma, assisted by thermal energy [2].

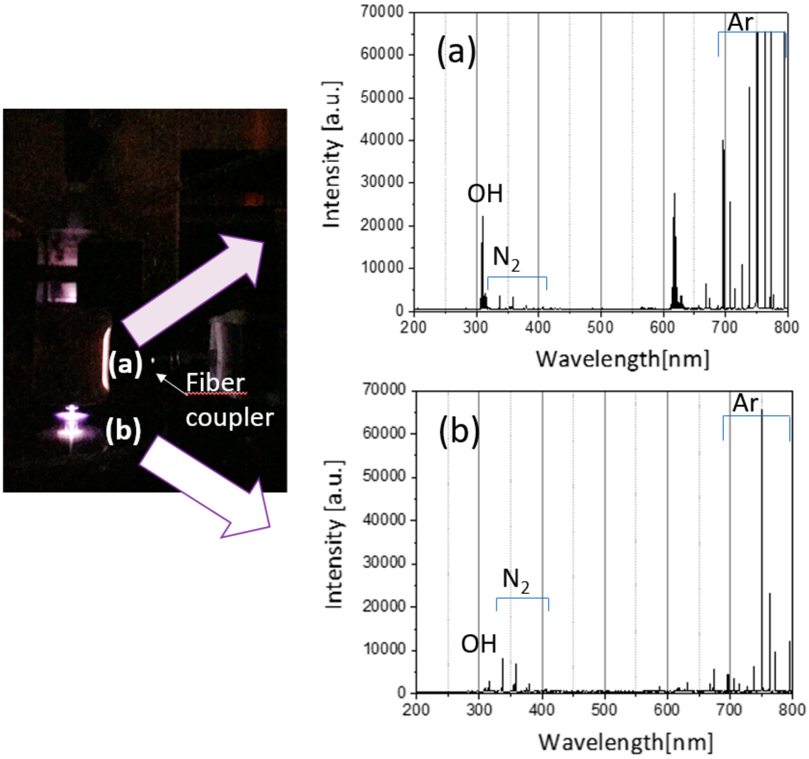

Figure S5. Optical emission spectra in the range of 200 – 800 nm, (a) in the active plasma discharge region; (b) near the substrate

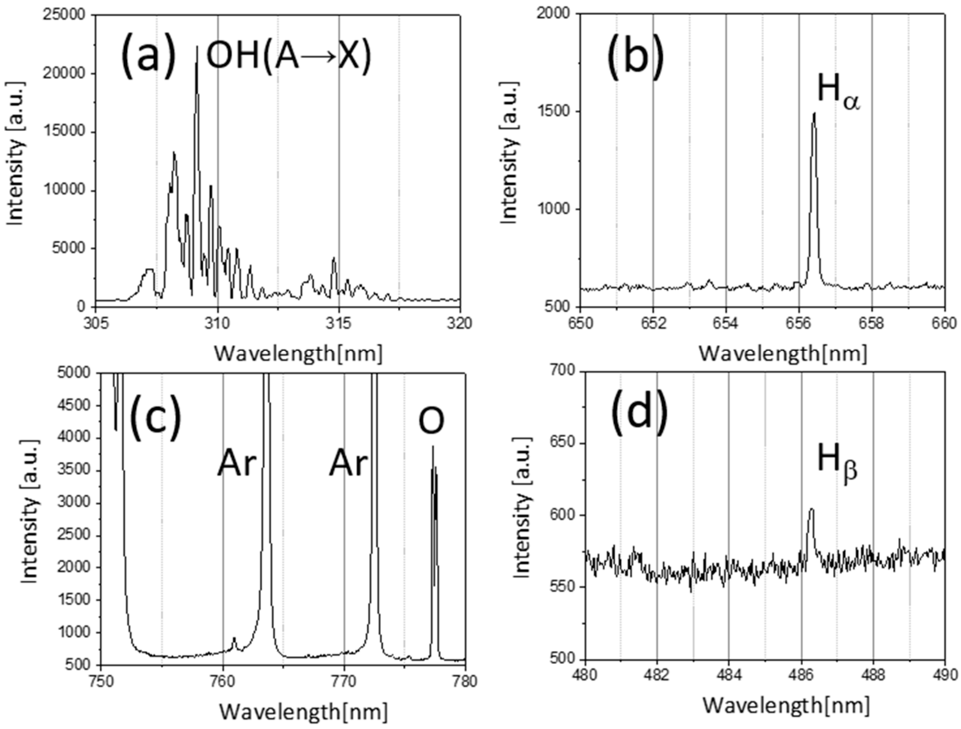

Figure S6. Optical emission from different species in the active discharge region (a) OH(A-X) (b) H_α (c) O and (d) H_β

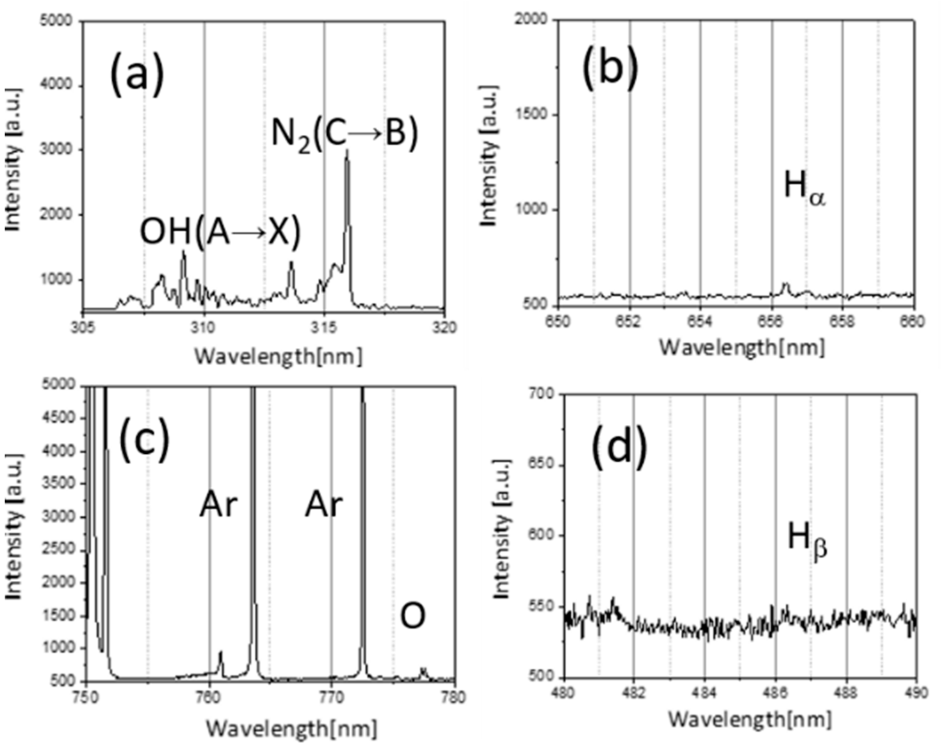

Figure S7. Optical emission from different species near the substrate (a) OH(A-X) (b) H_α (c) O and (d) H_β

Figure S6 shows the stronger emission of OH, atomic hydrogen and oxygen from the main discharge region in comparison to weak signal measured from near the substrate region as in Figure S7. The measurement condition of OES setting was the same. Electronic excitation of a molecule is generally predominantly governed by electrons. However, in case of OH(A-X) emission, it is well documented that dissociative excitation of water molecules by heavier molecules [3] dominates direct electron excitation from the ground state of OH(X). Argon ions and metastable argon atoms are known to have high reaction coefficients for the dissociative excitation of H_2O molecules [4, 5]. Both mechanisms, direct excitation from ground state by electrons or dissociative excitation by excited neutrals and ions, are more likely to occur in the active plasma zone between the electrodes, which is consistent with the higher OH emission intensity from this region.

The gas temperature is also a parameter that can have a significant influence on the plasma chemistry. The rotational temperature of the OH(A) state is often used to estimate the gas temperature in non-equilibrium plasmas, based on the assumption of efficient energy exchange between rotationally excited molecules and the background molecules [3]. However, especially when the precursor solution is introduced, the OH(A-X) spectrum becomes noisy. This leads to a significant fitting error to the spectra calculated by LIFBASE [6], which is an open-source code that simulates emission spectra of diatomic molecules, radicals and ions to investigate the rovibrational characteristics of the system. The estimated rotational temperature of

OH(A) states in an He-only plasma was relatively stable and calculated to be approximately 350 K for similar applied voltage and power to those used for the gold deposition process.

The surface temperature was monitored at the rear side of a glass substrate during stationary nanogold deposition using a thermistor (OPEGG WTS-3A-16). The temperature reached a maximum of 313 K. The estimated surface temperature on the top of the substrate was calculated to be 360 ± 30 K using the heat conduction equation, with the uncertainly corresponding to the possible error in estimates of the effective area and the delivered power. This value is close to the estimated value from the optical measurement. However, it should be stressed that the nanogold deposition on the paper base was done by scanning the plasma jet, so that the thermal energy delivered was very low.

4. Detailed SERS measurement results with Commercial SERS platform

Table S1. Cocaine bands detected in cocaine standards on the commercial P-SERS substrate.

**Bands listed in Ref. [3]**		**Commercial P-SERS substrate**							
Vibration Mode	Cocaine HCl salt	Cocaine HCl salt	5000 ng/mL	1000 ng/mL	500 ng/mL	100 ng/mL	10 ng/mL	1 ng/mL
(C-C) stretching (tropane ring)	848<br>874<br>898	853<br>870<br>897	-	-	-	-	-	-
Symmetric stretching-aromatic ring breathing	1004	1001	1002	1002	997	1004	999	997
Asymmetric stretching-aromatic ring breathing	1026	1027	1027	1030	1023	1025	1023	1023
C-N stretching	1165	1164	-	-	-	-	1162	-
C-N stretching	1207	1205	-	1198	-	-	-	-
Asymmetric CH_3 deformation	1462	1459	1446	-	-	-	-	-
C=C stretching-aromatic ring	1596<br>1601	1599	1603	1598	1591	1595	-	1594
C=O symmetric stretching-carbonyl	1716	1717	-	-	-	-	-	-

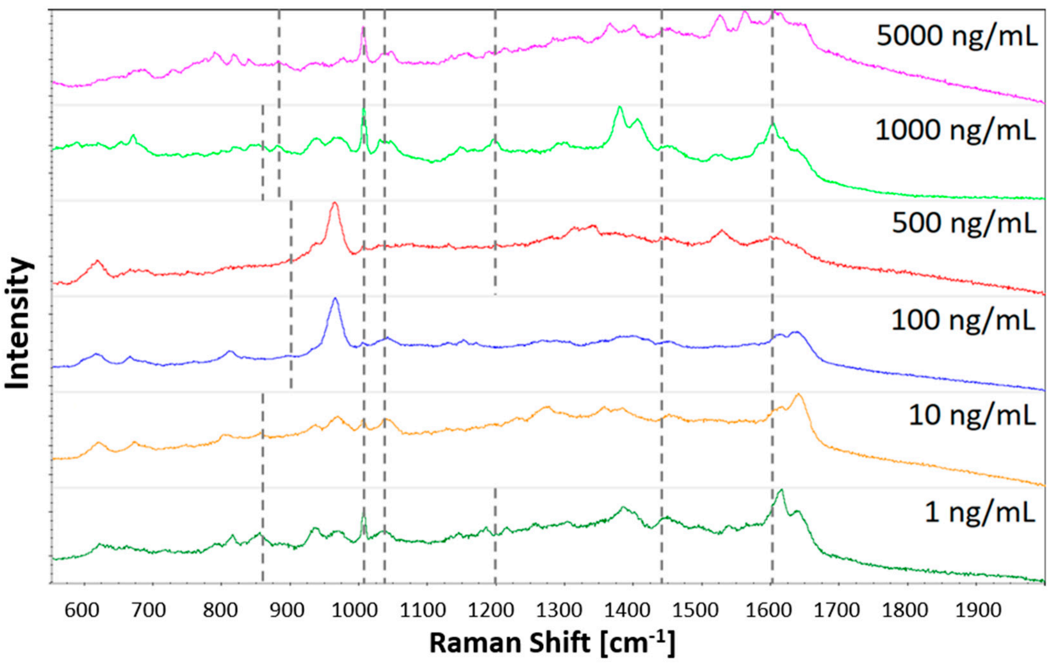

Figure S8. Comparison of SERS spectra acquired from standard cocaine solutions with decreasing concentrations deposited onto commercial JASMAT Ag. Reproduced from Alder et al. [7] with permission from John Wiley & Sons.

Table S2. Cocaine bands detected in cocaine standards on the JASMAT Ag substrate. Reproduced from Alder et al. [7] with permission from John Wiley & Sons.

Bands listed in Ref. [3]		JASMAT Ag substrate						
Vibration Mode	Cocaine HCl salt	Cocaine HCl salt	5000 ng/mL	1000 ng/mL	500 ng/mL	100 ng/mL	10 ng/mL	1 ng/mL
(C-C) stretching (tropane ring)	848 874 898	853 870 897	880	849 878	895	893	852	851
Symmetric stretching-aromatic ring breathing	1004	1001	1002	1003	1004	1002	1003	1003
asymmetric stretching-aromatic ring	1026	1027	1035	1027	1025	1038	1034	1034
C-N stretching	1165	1164	-	-	-	-	-	-
C-N stretching	1207	1205	1208	1195	1197	-	-	1212
Asymmetric CH_3 deformation	1462	1459	1448	1446	1446	1445	1448	1446
C=C stretching-aromatic ring	1596, 1601	1599	1603	1600	1596	1601	1601	1612
C=O symmetric stretching-carbonyl	1716	1717	-	-	-	-	-	-
C=O asymmetric stretching-carbonyl	1735	-	-	-	-	-	-	-

4. Cost of SERS substrate printing using plasma jet

The production cost of the plasma-printed nanogold SERS substrate is estimated and compared to the price of the two commercial strips in Table S3 as below.

Table S3. Comparison of SERS substrates showing nanoparticle type, backing substrate, deposition technique, particle size, size of active area and cost per substrate.

Substrate Name	Backing Substrate	Active Metal	Deposition Technique	Particle Size (nm)	Active Area Size (mm)	Cost per Substrate (AUD)
P-SERS	Paper	Gold	Inkjet printing [22]	70-85	9 x 5	~$10
JASMAT Ag	Silicon Pillars	Silver	e-beam evaporation	150-300 nm	3 x 3	$60-75
Plasma printed Substrate	Paper	Gold	Direct plasma printing	20-30	3 x 3	$0.20 (lab-scale without labour cost)

Based on current laboratory-scale system, the cost estimation of preparing a SERS substrate using the plasma jet system was obtained as below.

- Deposition area: 3 mm x 3 mm to allow comparison with commercial substrate
- Deposition time: 3 s x 6 passes = 18 s
- Electrical power consumption : 4 W in plasma discharge, estimated 10 W total power consumption
- Electricity price (domestic use) 0.255 AUD / kWh (1000 W x 3600 s = 3.6 MJ)
- AUD = Australian dollar

Table 4. Estimated cost of SERS strip printing using plasma jet based on current lab-scale system.

	Content	Cost per substrate
Electricity	18 s x 10 W = 180 J, 1.275 x 10-5 AUD	1.275 x 10-5 AUD
Gas	He 4 LPM<br>120 bar x 42 L -> 5040 L in a cylinder<br>350 AUD/cylinder<br>4 LPM x 18/60 min x 350 AU/5040 L = 0.08 AUD<br>Ar 1 LPM (assumed the same price as He)<br>0.02 AUD	AUD

Equipment	Depreciation of equipment based on 5-year lifetime<br>- Power supply 500 AUD<br>- Syringe pump 2000 AUD<br>- Mechanical parts 1000 AUD<br>- Mass flow controllers 2000 AUD<br>- Nebuliser 1000 AUD<br>Total 6500 AUD<br>1300 AUD/year<br>5.2 AUD/day (250 working days assumed)<br>0.65 AUD/h = 0.01 AUD/min (8 h/day)<br>0.003 AUD / 18 s for preparation of 1 substrate	0.003 AUD
HAuCl4	1% w/v, 20 μL/min (mixed 1:1 with ethanol)<br>25 g, 2560 AUD (Sigma Aldrich)<br>1 g/100mL for 1% w/v concentration<br>25 g is required for 2.5 L solution<br>2.5 L provides 8.3 x 105 samples of 3 μL each*<br>2560 AUD/8.3 x 105 substrates = 0.003 AUD/substrate<br>*20 μL/min x 0.5 (1:1 mixing) x 18/60 min<br>Need 3 μl for 1 substrate (3 mm x 3 mm)	0.003 AUD
Paper	Whatman filter paper<br>70 mm diameter, 100 pcs 35 AUD<br>π (3.5 cm)2 / [π (0.15 cm)2] =544 substrates possible on one filter paper<br>35 AUD/ 54,400 substrates = 6.4 x 10-4 AUD per substrate	6.4 x 10-4 AUD<br>< 0.001 AUD
Total	0.107 AUD	
Remark	To account for possible errors, the final cost was estimated as 0.2 AUD per substrate	

References

1. Hong, J.; Yick, S.; Chow, E.; Murdock, A.; Fang, J.; Seo, D.H.; Wolff, A.; Han, Z.; van der Laan, T.; Bendavid, A., et al. Direct plasma printing of nano-gold from an inorganic precursor. *Journal of Materials Chemistry C* **2019**, *7*, 6369-6374, doi:10.1039/C9TC01808E
2. Wu, T.-J., et al., *Ultrafast synthesis of continuous Au thin films from chloroauric acid solution using an atmospheric pressure plasma jet.* RSC Adv., 2015. **5**: p. 99654-99657.
3. Bruggeman, P.J., et al., *Gas temperature determination from rotational lines in non-equilibrium plasmas: a review.* Plasma Sources Science and Technology, 2014. **23**(2): p. 023001.
4. Bruggeman, P. and D.C. Schram, *On OH production in water containing atmospheric pressure plasmas.* Plasma Sources Science and Technology, 2010. **19**(4): p. 045025.
5. Novicki, S. and J. Krenos, *Absolute quenching cross section for collisions between Ar(3P0,2) and H2 O.* The Journal of Chemical Physics, 1988. **89**(11): p. 7031-7033.
6. Luque, J. and D.R. Crosley, *LIFBASE: Database and Spectral Simulation Program (Version 1.6).* SRI International Report MP, 99, (1999).
7. Alder, R.; Xiao, L.D.; Fu, S.L. Comparison of commercial surface-enhanced Raman spectroscopy substrates for the analysis of cocaine. Drug Test Anal 2020, 10.1002/dta.2894, doi:10.1002/dta.2894.
